# Dental and Skeletal Changes after Transpalatal Distraction

**DOI:** 10.1155/2020/5814103

**Published:** 2020-01-23

**Authors:** Ewa Zawiślak, Hanna Gerber, Rafał Nowak, Marcin Kubiak

**Affiliations:** Department of Maxillofacial Surgery, Silesian Piasts Medical University, Borowska 213, Wrocław 50-556, Poland

## Abstract

Maxillary constriction is a common skeletal craniofacial abnormality, and transverse maxillary deficiency affects 30% of patients receiving orthodontic and surgical treatment. The aim of the study was to analyse craniofacial skeletal changes in adults with maxillary constriction after transpalatal distraction. The study group consisted of 36 patients (16 women) aged 17 to 42 years (*M* = 27.1; SD = 7.8) with a known complete skeletal crossbite and who underwent transpalatal distraction procedure. The measurements were obtained on diagnostic models, and cephalometric PA radiograms were obtained at time points, i.e., before treatment (T1) and after the completion of active distraction (T2). The analysis of diagnostic models involving the arch width measurement at different levels demonstrated a significant increase in L1, L2, L3, L4, L5, and L6 dimensions after transpalatal distraction. The largest width increase (9.5 mm) was observed for the L3 dimension (the intercanine distance). The analysis of frontal cephalograms displayed a significant increase in W1, W2, and W3 dimensions after transpalatal distraction. The largest width increase (4.9 mm) was observed for the W1 dimension at the level of the alveolar process of the maxilla. Transpalatal distraction is an effective treatment for transverse maxillary deficiency after the end of bone growth. The expansion observed on diagnostic models is close to a parallel segment shift mechanism, with a mild tendency towards a larger opening anteriorly. The maxillary segment rotation pattern analysed based on the frontal cephalograms is close to a hand fan unfolding with the rotation point at the frontonasal suture.

## 1. Introduction

Maxillary constriction is a common skeletal craniofacial abnormality. 30% of patients receiving any complex orthodontic and surgical treatment suffer from transverse maxillary deficiency [1]. The appropriate transverse dimension of the upper arch ensures stable occlusion and significantly affects facial proportions and aesthetics [2, 3]. Clinically, transverse maxillary deficiency manifests as a complete crossbite (unilateral or bilateral), high-vaulted, V-shaped palate, with anterior tooth crowding and visible buccal corridors when smiling [4–7]. Laptook identified core clinical features of “skeletal malocclusion syndrome” if a complete crossbite is present, which include increased nasal breathing difficulty, reduced nasal cavity volume, mouth breathing, crossbite with a high-vaulted hard palate, and turbinate hypertrophy. Presence of at least two of the aforementioned clinical features indicates skeletal malocclusion and necessitates treatment aimed at increasing the transverse maxillary dimension and its skeletal expansion [8].

Using orthodontic expanders for the treatment of maxillary deficiency in adults leads to a number of dental and periodontic complications [7, 9–13]. By acting indirectly on the midpalatal suture, the tooth-borne appliances adversely affect lateral teeth, causing buccal inclination and extrusion, alveolar bone fenestration along its buccal aspect, dental root resorption, and gingival recession in the proximity of the teeth on which the appliance is borne [9]. The adverse effects of such appliances on periodontium worsen with the patient's skeletal maturity [14].

Due to the limitations of dental anchorage in the rapid maxillary expansion (RPE) in adult patients, the skeletal anchorage was introduced in the method of microimplant-assisted rapid palatal expansion (MARPE), as an alternative to surgically assisted jaw expansion modalities. MARPE involves the use of a hybrid device: bone anchored with 2 mini-implants on each side of the midpalatal suture and tooth-borne on the first upper molars [15].

Midface anatomy and architecture are key aspects of maxillary expansion [16–18]. The flexibility of the bone skeleton decreases with age, which limits the possibility of orthodontic and orthopaedic treatments, therefore making surgical intervention a necessity [19, 20]. When performed within the midface, LeFort I osteotomy enables maxillary expansion whilst minimising stress to other craniofacial regions and the skull base [17, 21–23]. The literature, however, does not specify unequivocally the age limit, up to which orthodontic maxillary expansion is effective and free of complications. Most authors point towards 14–18 years of age as the upper limit of any orthodontic midpalatal interventions [7, 24–27].

Surgical maxillary expansion is in fact limited to two surgical techniques of segmented maxillary osteotomy and transpalatal distraction (TPD) and their modifications [28]. The first method allows for a simultaneous increase of the maxillary transverse dimension and maxillary correction in other planes. The disadvantages of a segmented maxillary osteotomy include surgical complexity, a limited degree of maxillary expansion due to relative stiffness and inflexibility of palatal mucosa (up to 5 mm stretch is only possible), and the risk of recurring unstable occlusal relationships [13, 29]. Transpalatal distraction of severe maxillary deficiency in adults was introduced by Mommaerts in 1999 [5–7]. The procedure is performed under general anaesthesia and involves a subtotal LeFort I maxillary osteotomy and transpalatal suture mobilisation. The source of expansive force in this method is a bone-borne transpalatal distractor anchored to the hard palate [30–35].

## 2. Materials and Methods

The study group consisted of 36 patients (16 women) aged 16 to 49 years (*M* = 27.1; SD = 7.8) with a known complete skeletal crossbite and who underwent a transpalatal distraction procedure. All patients were treated at the Chair and Department of Maxillofacial Surgery, Medical University in Wrocław, between 2012 and 2015.

### 2.1. Surgical Procedure

**Figure 1:**
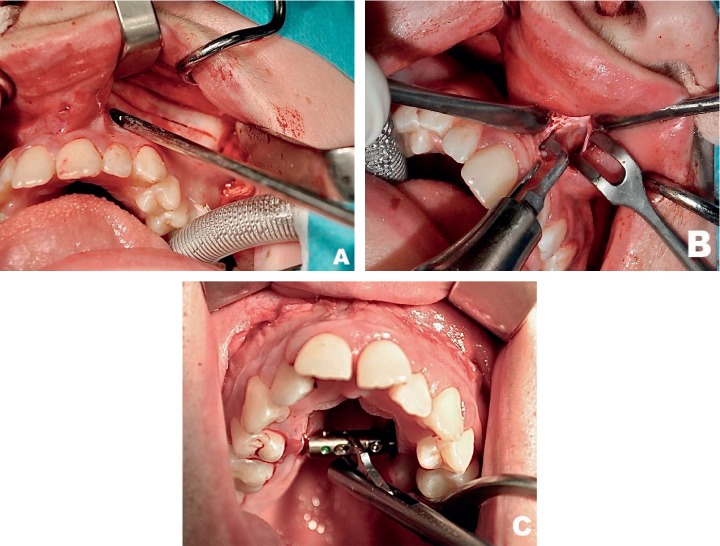
Intraoperative view. (a) Transverse osteotomy line. (b) Midpalatal osteotomy. (c) Fitted bone-borne transpalatal distractor.

A subtotal maxillary osteotomy (LeFort I) with the separation from the pterygoid process of the sphenoid and sagittal midpalatal suture osteotomy was performed under general anaesthesia, with orotracheal intubation and antibiotic prophylaxis. The procedure ended by fitting the transpalatal distractor (Unismile, Titamed, Belgium) anchored to the hard palate at the premolar level (Figure 1). It was activated intraoperatively until it achieved a diastema sized 0.5–1.0 mm. The distraction was continued by expanding the distractor by 0.25 mm twice a day for a period depending on the degree of transverse maxillary deficiency at baseline. The follow-up appointments during the active distraction period were scheduled every 7 days. The active distraction was considered complete after the planned arch width had been achieved. Subsequently, a maxillary diagnostic model and radiograms (including a pantomogram and frontal cephalogram) were repeated.

### 2.2. Diagnostic Model Analysis

**Figure 2:**
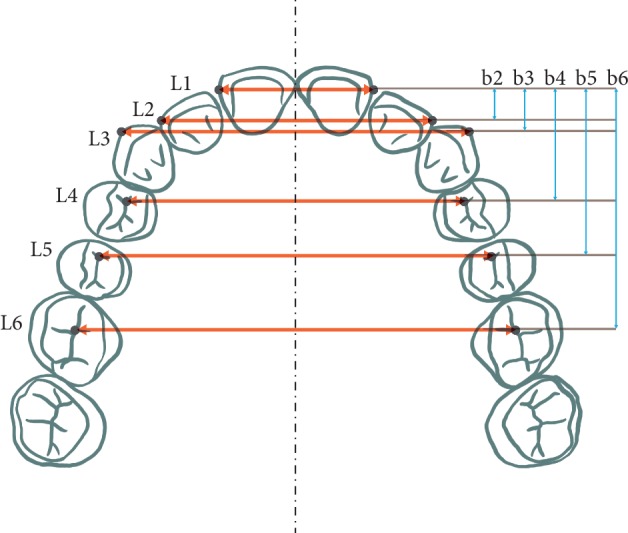
Reference points and respective measurement segments on maxillary diagnostic models.

All measurements were taken on diagnostic models cast using the conventional method at two time points: before treatment (T1) and after the completion of active distraction (T2). All measurements were made using a manual caliper, whilst free of eye strain and fatigue. To determine the maxillary dental arch widths, the authors chose the measurement points corresponding to individual maxillary teeth and assumed six measured distances (segments). The reference points and segments measured on the models are shown in Table 1. The second measurement of b2, b3, b4, b5, and b6 distances (segments) was performed using AutoCAD LT after the pretreatment (T1) and posttreatment (T2) model photographs had been taken. Additional measurement of the b2, b3, b4, b5, and b6 distances was aimed at graphic presentation of maxillary expansion on the diagnostic models (Figure 2).

### 2.3. Analysis of Frontal PA Cephalograms

**Figure 3:**
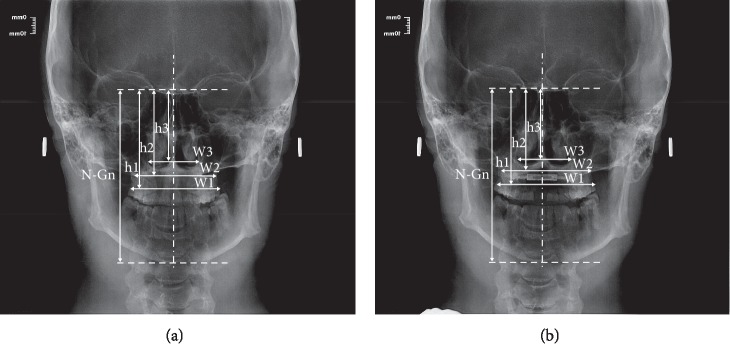
(a) Distances (segments) measured in frontal cephalometric radiograms prior to treatment (T1). (b) Distances (segments) measured in frontal cephalometric radiograms after the completion of active distraction (T2).

The frontal cephalometric PA radiogram was carried out using the Kodak CS9000 digital X-ray device. The respective measurement values were analysed using the VixWin Platinum 3.0 (Gendex) software bundle. Distance calibration was performed prior to the actual measurement. The reference points and segments were introduced, which determined the maxillary alveolar width (MAW), maxillary base width (MBW), and nasal width (NW). The following vertical measurement segments were determined: N-Gn and h1, h2, and h3. The N-Gn segment was determined based on the respective craniofacial bone landmarks. The h1 segment was defined as the distance between the nasion point and W1 segment so that they cross at a right angle. Accordingly, the h2 segment was defined as the distance between the nasion point and the W2 segment so that they cross at a right angle, and the h3 segment was defined as the distance between the nasion point and the W3 segment so that they cross at a right angle. All reference points and segments in the frontal cephalometric radiograms are shown in Table 2. Each measurement was taken at two time points: prior to treatment (T1) and after the completion of active distraction (T2) (Figures 3(a) and 3(b)).

## 3. Results and Discussion

The paired-sample *t*-test confirmed significant changes in the mean length of all segments (L1–L6 and W1–W3) at the end of active distraction (T2) from baseline (T1) (*p* < 0.05). Other measurements, except for h2, did not significantly change from baseline (*p* > 0.05). The results are shown in Table 3.

**Figure 4:**
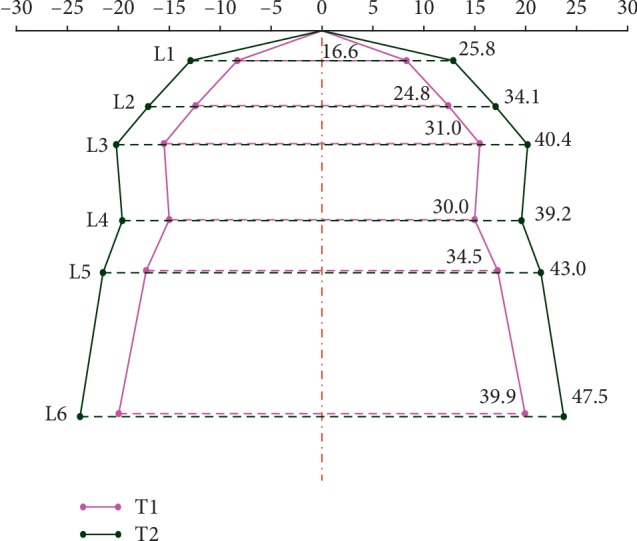
Graphic representation of the change prior to treatment (T1) at the completion of active distraction (T2) on maxillary diagnostic models.

**Figure 5:**
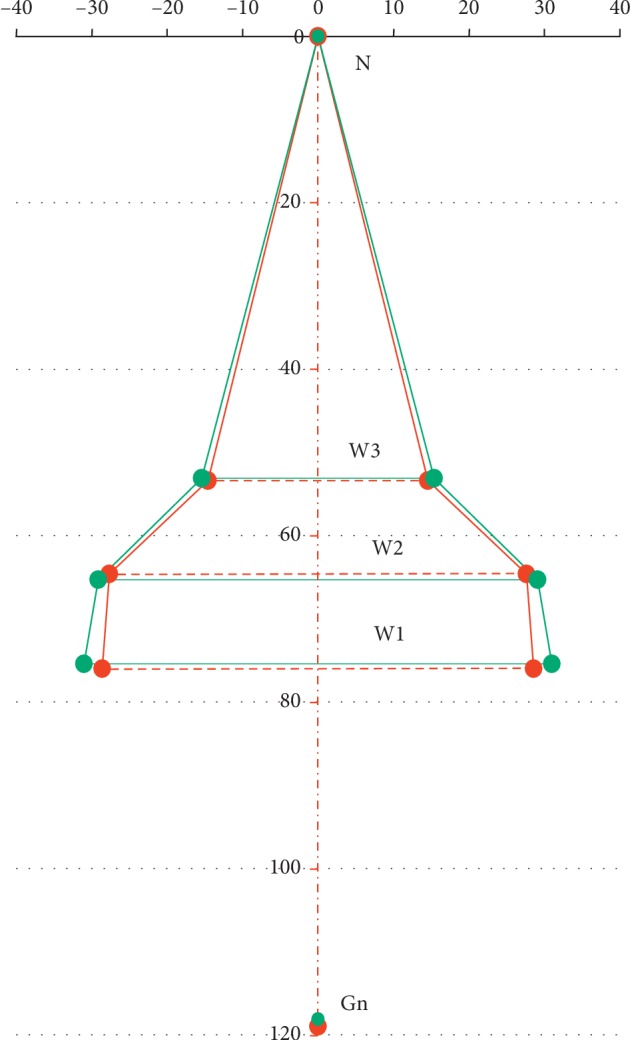
Graphic representation of the change prior to treatment (T1) at the completion of active distraction (T2) in frontal cephalometric radiograms.

The change from baseline (T1) at the end of treatment (T2) is presented graphically on diagnostic models (Figure 4) and in frontal cephalometric radiograms (Figure 5).

The analysis of diagnostic models involving the arch width measurement at different levels demonstrated a significant increase in L1, L2, L3, L4, L5, and L6 dimensions after transpalatal distraction in patients with transverse maxillary deficiency. Six measurement segments (distances) were used, each corresponding to a separate tooth group. The aim was to characterise the pattern of maxillary segment expansion in an axial plane. The mean distances of the L1 dimension (at the medial incisor level) increased by 9.3 mm, L2 (at the lateral incisor level) by 9.2 mm, L3 (at the canine level) by 9.5 mm, L4 (at the first premolar level) by 9.3 mm, L5 (at the second premolar level) by 8.6 mm, and L6 (at the first molar level) by 7.6 mm. The results of these measurements are in line with the findings reported by Matteini and Mommaerts [6], who described parallel distraction and shift of maxillary segments along the axial plane if the maxilla has separated from the pterygoid process of the sphenoid and the distractor has been fitted more distally [6, 7, 36, 37]. Ramieri et al. analysed maxillary diagnostic models in 29 patients after transpalatal distraction as per Mommaerts' protocol. They measured 3 arch dimensions: (1) arch width at the level of the first molars, (2) arch width at the level of the first premolars, and (3) arch width at the level of the canines, noting the largest increase (7.29 mm) at the level of the canines, followed by the first premolars and the first molars (6.86 mm and 5.39 mm, respectively). Furthermore, they compared outcomes between patients after a maxillary expansion with and without complete pterygomaxillary separation. The arch width increased by 6.83 mm in a group after a complete pterygomaxillary separation compared to 6.81 mm in a group after an incomplete osteotomy [4]. This shows that a pterygomaxillary separation did not significantly affect the degree of anterior maxillary expansion. Posteriorly though, the expansion patterns differed. At the level of the molars, the arch width increased by 6.47 mm in a group after a complete pterygomaxillary separation compared to 4.42 mm in a group after an incomplete osteotomy. It is a commonly held view that the osteotomy type in both SARPE and TPD should be determined based on the original arch shape and degree of transverse maxillary deficiency [4, 5, 36]. Günbay et al. reported an increase in the transverse maxillary dimension by 5.00 mm, 5.99 mm, 6.10 mm, 7.07 mm, 7.10 mm, and 6.10 mm at the level of medial incisors, lateral incisors, canines, first premolars, second premolars, and first molars, respectively, in patients with transverse maxillary deficiency treated using a bone-borne maxillary expander [2]. They observed an irregular maxillary expansion pattern and attributed it to buccal inclination of the premolars, which corresponded to the transpalatal distractor fixation site. Laudemann et al. explained the largest maxillary expansion at the level of premolars by the direct application of the expansive force at this particular site and the presence of the strongest resistance force in the posterior part of the maxilla [38, 39]. Matteini and Mommaerts described two distinct maxillary expansion patterns in patients after TPD, i.e., anterior and posterior distraction, with the latter also known as parallel distraction [6, 7]. The anterior distraction is characterised by the largest increase in arch width in the anterior segment of the V-shaped arch with the PNS (posterior nasal spine) point being the centre of rotation along the horizontal plane. This expansion pattern results from the resistance at the pterygomaxillary junction, which was confirmed in the finite element method studies [5–7, 17, 26, 27]. In contrast, parallel distraction is characterised by an equal arch width increase in both its anterior and posterior aspects. This expansion pattern follows a complete pterygomaxillary separation (disjunction) which enables equal distribution of expansive force between the two maxillary segments [22, 26, 27, 40, 41].

Vertical dimensions (b2, b3, b4, b5, b6) were not used in analysing diagnostic maxillary models. We decided to introduce the vertical measurements in diagnostic model photographs using the AutoCAD LT bundle, in order to enable a graphic representation of maxillary expansion pattern after a complete TPD. The analysis of frontal cephalometric radiograms showed a significant increase in W1, W2, and W3 dimensions in patients after transpalatal distraction. The largest amount of width increase (4.9 mm) was observed for the W1 dimension, at the level of the alveolar process of the maxilla. The W2 (base of the maxilla) and W3 (pyriform aperture at the base of the inferior concha) dimensions increased by 2.9 mm and 1.7 mm, respectively. The vertical dimensions (N-Gn, h1, h2, and h3) were not used in the transverse maxillary assessment. They were introduced in order to enable a graphic representation of maxillary expansion pattern in frontal cephalograms after a complete TPD. Our findings in this regard are in line with the published results [2, 9, 16, 42, 43]. Günbay et al. reported the mean arch width increase after transpalatal distraction by 7.75 mm, 5.25 mm, and 4.3 mm, at the level of the alveolar process, maxillary base, and nasal base, respectively, measured in the frontal cephalograms [2].

The observed increase in the W3 dimension corresponds to patient-reported improvement. The W3 dimension was the width at the pyriform aperture at the base of the inferior concha. Although treatment-induced changes to the nasal cavity volume were not an endpoint of this study, all patients reported improved nasal patency after treatment. Aras et al. evaluated dimensional changes of the nasal cavity in 11 adults after TPD with a bone-borne distractor, using acoustic rhinometry (AR) and computed tomography (CT). The analysis of changes in transverse dimensions using CT scanning showed significant expansion of nasal cavity with decreasing magnitude from alveolar level to nasal cavity base. The increase of these dimensions was 4.68 mm and 2.73 mm at the canine region and at the level of first molars, respectively. The acoustic rhinometry demonstrated significant increases in volumes of nasal cavity by 101% in the anterior nasal cavity and 120% in the posterior nasal cavity [12, 44, 45]. Seeberger et al. evaluated changes after SARPE in 31 patients with maxillary constriction based on cone-beam tomograms. They used the SimPlant® OMS (v12.03, Materialise Co, Leuven, Belgium) and Mimics® (v12.3, Materialise Co, Leuven, Belgium) software bundles to analyse the changes in different interdental arch widths and changes to lower nasal airway assessed from anterior to posterior. The mean distraction width was 6.5 mm [46]. The distraction width values of the nasal floor indicated a larger amount of expansion anteriorly (2.5 mm) than posteriorly (0.97 mm) which was described as a V-shaped opening [2, 13, 37, 45, 47].

## 4. Conclusions

1. Transpalatal distraction is an effective treatment of transverse maxillary deficiency after the end of bone growth
2. Active distraction requires close monitoring by the MDT consisting of an orthodontist and a maxillofacial surgeon
3. Direct measurements on maxillary diagnostic models demonstrate arch width increase at all measured levels
4. The largest expansion was demonstrated by the increase of the intercanine distance (L3 dimension)
5. Indirect measurements in frontal PA cephalograms demonstrated increased arch width at all measured levels
6. The largest amount of width increase was shown for the W1 dimension, at the level of the alveolar process of the maxilla, and the smallest amount of width increase was shown for the W3 dimension, at the nasal base
7. The expansion observed on diagnostic models is close to a parallel segment shift mechanism, with a mild tendency towards a larger opening anteriorly
8. The maxillary segment rotation pattern analysed based on frontal cephalograms is close to a hand fan unfolding with the rotation point at the frontonasal suture

## Figures and Tables

**Table 1:** Presentation and description of reference points on maxillary diagnostic models.

Reference point	Distance
Distal surface of maxillary medial incisors	L1
Distal surface of maxillary lateral incisors	L2
Maxillary canine cusp	L3
Central groove midpoint of the first maxillary premolars	L4
Central groove midpoint of the second maxillary premolars	L5
Central pit of the first maxillary molars	L6

**Table 2:** Reference points and their respective segments in frontal cephalograms.

Reference point	Segment
Lateral border of the pyriform aperture at the base of the inferior concha	W3
Lateral maxillary wall at the base of the zygomaticoalveolar crest	W2
Buccal surface of the second maxillary molar	W1

**Table 3:** Descriptive statistics and within-subject comparisons of measurements before (T1) and after treatment (T2).

Measurements	Pretreatment (T1) (MeanSD)	Posttreatment (T2) (MeanSD)	*p* value
L1	16.6	25.8	<0.0001
L2	24.8	34.1	<0.0001
L3	31.0	40.4	<0.0001
L4	30.0	39.2	<0.0001
L5	34.5	43.0	<0.0001
L6	39.9	47.5	<0.0001
b2	4.5	4.6	0.472
b3	8.3	8.4	0.429
b4	15.9	16.0	0.385
b5	21.0	21.2	0.318
b6	35.3	35.6	0.339
W1	57.2	62.0	<0.0001
W2	55.3	58.2	<0.0001
W3	29.1	30.8	<0.0001
N-Gn	119.0	118.1	0.394
H1	43.0	42.7	0.247
H2	54.4	52.8	0.002
H3	65.6	65.0	0.203

SD, standard deviation.

## Data Availability

The clinical data used to support the findings of this study are available upon request to the corresponding author Ewa Zawiślak (Ewazawislak0@op.pl).
